# Who’s on Call? Mandibular Fracture Management at a Level I Trauma Center

**DOI:** 10.3390/jcm14134707

**Published:** 2025-07-03

**Authors:** Allyson R. Alfonso, Maxime M. Wang, Alexis K. Gursky, Hailey P. Wyatt, Jonathan M. Bekisz, Karl Bruckman, Spiros G. Frangos, Pierre B. Saadeh

**Affiliations:** 1 Bellevue Hospital Center, New York City, NY 10016, USA; 2 Hansjörg Wyss Department of Plastic Surgery, NYU Langone Health, 222 East 41st Street, 7th Floor, New York, NY 10017, USA; 3 Division of Plastic and Reconstructive Surgery, Stanford University School of Medicine, Stanford, CA 94305, USA; 4 Division of Trauma & Surgical Critical Care, Department of Surgery, NYU Langone Health, New York, NY 10017, USA

**Keywords:** mandibular fracture, facial fracture, facial trauma, complications, management, outcomes

## Abstract

**Background**: Facial trauma is one of the few surgical conditions that is routinely managed by three distinct disciplines, including Oral and Maxillofacial Surgery (OMS), Plastic and Reconstructive Surgery (PRS), and Otolaryngology (ENT). This study aims to evaluate mandibular trauma management strategies and clinical outcomes among three operating services. **Methods**: An IRB-approved, retrospective chart review was performed over a ten-year period (2007–2016) at a major, urban, Level I trauma center for all patients treated for an isolated mandibular injury determined by ICD-9 codes. Of the 2299 patients evaluated for traumatic facial injuries, 191 met the inclusion criteria and 137 had longitudinal data. Patient, fracture, and management characteristics and clinical outcomes were compared among three surgical services. **Results**: Most patients were male (95.3%), and assaults were the most common etiology of injury (79.1%). The angle/ramus was the most common single location (31.4%), and 47.6% of patients had multiple fractures. There was a statistically significant difference between specialties when assessing the use of operative versus non-operative approaches to fracture management (*p* < 0.001), and within operative management, for the use of open reduction-internal fixation (ORIF) alone versus ORIF with maxillomandibular fixation (MMF) (*p* = 0.002). There was no significant difference in the overall complications between specialties (*p* = 0.227). **Conclusions**: Services differ in their decision to pursue operative versus non-operative management, as well as the decision for postoperative MMF, though these differences in decision-making were not associated with a significant difference in the overall complications.

## 1. Introduction

The mandible is one of the most commonly fractured bones of the facial skeleton [1,2]. Frequently encountered in males in their third decade of life, patients typically present in the acute phase after an assault, fall, or motor vehicle collision [3]. Mandibular fractures can occur in isolation or with other facial fractures and may involve one or numerous sites on the mandible. Ideal management of these fractures results in restoration of both the mandibular structure and function by providing a good bony reduction with adequate soft tissue coverage, as well as re-establishing normal dental occlusion and temporomandibular joint (TMJ) alignment [2].

Various approaches to the treatment of mandibular fractures exist. Many factors affect a surgeon’s decision regarding operation, incisional approach, and method of fixation. The choice of non-operative, closed reduction with or without maxillomandibular fixation (MMF) versus operative reduction with internal fixation (ORIF) may be affected by factors such as extent and location of fractures, fracture displacement, dental malocclusion, medical comorbidities, and patient compliance [2,4]. Surgical management of fractures may employ varying incisional approaches and different configurations of hardware by which to achieve two points of fixation [2]. Interestingly, various institutions have established facial trauma as one of the few surgical complaints that is routinely managed by one of three distinct disciplines, including Oral and Maxillofacial Surgery (OMS), Plastic and Reconstructive Surgery (PRS), and Otolaryngology (ENT) [5].

Given the diversity of fracture patterns and treatment algorithms, reported rates of complications in the treatment of mandibular fractures vary greatly (4.2–53%) [6,7,8]. Complications of mandibular fractures include postoperative infection (5.7–6.95%), bony nonunion (1–2.2%), nerve injury, malunion, and TMJ dysfunction [6,9]. Fracture characteristics [6,7,8], management characteristics [6,9], and patient characteristics, such as substance abuse and chronic medical conditions, have been shown to affect complication rates to varying degrees [9,10,11,12]. Previously, studies have compared the outcomes of facial fractures in cases managed by plastic surgery compared to other surgical services, and no significant association has been demonstrated between the operating service and complication rates [5,13]. The studies have been limited to an analysis of 30-day postoperative data available in national surgical databases that can provide only a cursory assessment of mandibular fracture management outcomes between sub-specialties [5,13]. Leveraging our institutional experience with a consistent rotating facial trauma call schedule between OMS, PRS, and ENT, as well as stable faculty retention, and long-term, clinically relevant follow-up, we aimed to design the most comprehensive institutional study to date to compare the management of isolated mandibular fractures between operating services. We also sought to assess the effect of potential differences in management strategies on clinically significant outcomes in an effort to inform standardized practices in the treatment of this potentially challenging surgical problem.

## 2. Materials and Methods

This was an Institutional Review Board-approved, 10-year retrospective chart review (2007–2016) performed at a large Level I trauma and tertiary care center. We pooled patients from the trauma surgery service database using the following ICD-9 codes: 802.2, 802.21, 802.22, 802.24, 802.25, 802.26, 802.28, 802.29, 802.3, 802.31, 802.32, 802.34, 802.35, 802.36, 802.38, and 802.39. Inclusion criteria were those patients documented as having isolated mandibular fractures. Exclusion criteria were those patients with additional facial fractures or patients with inadequate record keeping [14,15]. A total of 2299 patients with facial fractures were identified, 191 of which had isolated mandible fractures and met the inclusion criteria. A minimum sample size of 108 patients was needed based on a power analysis for a Chi-square test of independence (Cohen’s w = 0.3, α = 0.05, power = 0.80, df = 2). Demographic data and operative and non-operative management of these patients were compared amongst the three surgical services commonly treating these injuries (OMS, PRS, and ENT). Our study variables included demographic patient information, injury type, fracture classification—including displacement if at least one fracture was displaced—or comminution if at least one fracture was comminuted, overall fracture management, hospital quality indicators (length of stay, 30-day readmission, length of follow-up), and complications documented for the duration of follow-up. Variables were defined as being present when explicitly documented as such, and absent when documented as non-contributory or not documented. Major psychiatric illness and substance use disorder were defined according to the Diagnostic and Statistical Manual of Mental Disorders, Fifth Edition (DSM-5) diagnostic criteria. Major psychiatric illness encompassed major depressive disorder, bipolar I or II disorder, or schizophrenia. Substance use disorder refers to moderate to severe use of alcohol or illicit drugs, characterized by impaired control, physical dependence, social dysfunction, or risky use. Injury Severity Score, a quantifier for extent of injury, was recorded for each patient at the time of injury and available in the trauma registry [16,17]. Information on long-term complications was present for 137 out of 191 patients. Only patients with post-discharge data were included in this analysis, as one aim was to assess long-term clinical outcomes between specialties [18]. The overall complication rate was determined by the presence of any documented complication (new malocclusion, aesthetic deformity, temporomandibular joint (TMJ) dysfunction, new nerve injury (paresthesia), hardware failure, dental injury, chronic pain, wound complication, and infection). Wound complication specifically refers to wound dehiscence, hypergranulation, and/or wound-related infection, while overall infection included wound, hardware, and systemic infection [19].

Statistical analysis was conducted using a one-way analysis of variance (ANOVA) for parametric variables [20]. Tukey’s and Games–Howell post hoc tests were used for equal and unequal variances, respectively. The Kruskal–Wallis Test was used for ordinal, non-parametric variables, and Chi-square or Fisher’s exact test (*n* < 5) were used accordingly for nominal, non-parametric variables [21]. For contingency tables comparing the three specialties, post hoc analyses were conducted using standardized residuals to identify cell-level contributions to significant Chi-square associations [22]. For residuals with an absolute value greater than 2.00 (|Z| > 2.00), the corresponding Chi-square statistic (Z^2^) and *p*-value were calculated, applying a Bonferroni correction to account for a type I error across multiple comparisons [23]. Data analyses were performed using SPSS version 23.0 (IBM Corp., Armonk, NY, USA) for ANOVA with post hoc analyses, Kruskal–Wallis, Chi-square, and Fisher’s exact test (2 × 2 contingency tables only). RStudio 3.3.3 was used for Fisher’s exact tests with analyses larger than a 2 × 2 contingency table (R Foundation for Statistical Computing, Vienna, Austria) [24].

## 3. Results

### 3.1. Patient Characteristics

A total of 191 patients with isolated mandible fractures met the inclusion criteria. Table 1 depicts the characteristics of these patients. The majority of the patient population was male (95.3%), with an ASA classification lower than 3 (95.3%). Assaults were the most common etiology of injury (79.1%). The OMS service saw the most clinical cases of isolated mandibular fracture (45.0%). There was no significant difference between specialties for patient age (OMS 32.7 vs. PRS 28.0 vs. ENT 30.0 years, *p* = 0.09), gender (*p* = 0.68), Injury Severity Score (*p* = 0.97), ASA classification (*p* = 0.51), smoking history (*p* = 0.36), or any of the documented comorbidities, including major psychiatric illness (*p* = 0.69), asthma (*p* = 0.18), substance use disorder other than alcohol use (*p* = 0.19), cardiovascular disease including hypertension (*p* = 0.42), alcohol use disorder (*p* = 0.47), and diabetes (*p* = 0.09).

### 3.2. Fracture Characteristics

As delineated in Table 2, 47.6% of patients with mandibular trauma presented with fractures in more than one location. Of the single mandible fractures, the most common location was the angle/ramus (31.4%). There was a significant difference in fracture location between specialties (*p* = 0.025), where ENT treated more parasymphyseal fractures (13.7%) than OMS (1.2%) and PRS (3.7%) (*p* = 0.001).

Displaced fractures comprised 55.0% of all cases, with no significant difference in distribution across specialties, χ^2^(2, *N* =191) = 0.888, *p* = 0.64. They were observed in 58.1% of OMS patients (*n* = 50), 54.0% of PRS (*n* = 27), and 51.0% of ENT (*n* = 28). Comminuted fractures represented 18.8% of the cohort, and were similarly distributed among specialties, χ^2^(2, *N* =191) = 0.869, *p* = 0.65. These accounted for 19.8% of OMS cases (*n* = 17), 14.8% of PRS (*n* = 8), and 18.8% of ENT (*n* = 11). Similarly, the proportion of patients with multiple versus single mandibular fractures (47.6% multiple) did not differ by specialty, χ^2^(2, *N* =191) = 5.092, *p* = 0.08. There was a significant difference in treatment of open versus closed fractures, χ^2^(2, *N* =191) = 11.87, *p* = 0.003, where ENT treated significantly more open fractures (*n* = 16, 31.4%) than OMS (*n* = 10, 11.6%) and PRS (*n* = 5, 9.3%; *p* = 0.001), and significantly less closed fractures (*p* = 0.001). When multiple fractures were stratified, there was a total of 291 fractures, with fractures of the angle/ramus being the most common (43.6%), and with no significant difference between specialties (*p* = 0.262) (Table 3).

The OMS service was associated with significantly more (56.3%) and ENT with significantly less (19.3%) documentation of malocclusion at the initial encounter between all three specialties (PRS 24.4%; *p* < 0.001).

### 3.3. Fracture Management

There was a statistically significant difference between specialties when assessing the utilization of operative compared to non-operative approaches to fracture management, χ^2^(2, *N* =191) = 18.42, *p* < 0.001, with the OMS service using more operative management (88.4%), followed by PRS (79.6%), and ENT using non-operative management significantly more often (43.1%; *p* < 0.001). There was a significant difference between specialties for fracture location treated operatively (*p* = 0.002) (Table 4). Within the group of fractures treated operatively, ENT treated significantly more parasymphyseal fractures (*n* = 6) than OMS (*n* = 1) and PRS (*n* = 1; *p* < 0.001).

There was no significant difference in the distribution of fracture location between specialties for those treated non-operatively. There was a significant difference in the decision for tooth extraction between specialties, with OMS performing the most tooth extractions (OMS 41.9% vs. PRS 7.4% vs. ENT 0.0%; *p* < 0.001).

When assessing operative management (Table 5), there was a significant difference between specialties for use of ORIF alone versus ORIF with MMF, χ^2^(2, *N* =148) = 12.42, *p* = 0.002. Post hoc analysis showed that ENT was more likely to use ORIF with MMF (65.5%) than OMS (30.3%) and PRS (30.2%; *p* < 0.001). There was no significant difference in operative approach (transoral, transfacial, combination) between specialties (*p* = 0.211), though overall the transoral approach was most commonly used across specialties (83.0%). One patient was excluded from this analysis only, as there was no operative report available for review. There was no significant difference between specialties for number of plates used per fracture overall (1.2 ± 0.52, *p* = 0.230).

Irrespective of management approach, there was no significant difference between specialties for the rate of thirty-day readmission (2.1%, *p* = 0.288). Length of stay significantly differed between specialties (*p* = 0.017), with PRS having a significantly longer length of stay than ENT (4.8 ± 3.8 vs. 3.1 ± 2.9 days; *p* = 0.036), but no difference to OMS (4.1 ± 2.2 days; *p* > 0.100).

### 3.4. Complications

The inclusion criteria for complications and follow-up was met by 137 patients who had clinical data available following hospital discharge. The length of follow-up for patients was not significantly different between specialties (*p* = 0.899). The median length of follow-up was 49.0 days, with a 95% confidence interval ranging from 43.0 to 58.0 days. The overall complication rate for mandibular fractures was 37.2%, including new malocclusion, aesthetic deformity, temporomandibular joint (TMJ) dysfunction, new nerve injury, hardware failure, dental injury, chronic pain, wound complication, and infection. There was no significant difference in the overall complications between specialties, χ^2^(2, *N* =137) = 2.97, *p* = 0.227, (OMS 44.3% vs. PRS 27.0% vs. ENT 35.9%). When stratified by complication type, there was no significant difference between specialties for new malocclusion, aesthetic deformity, TMJ dysfunction, new nerve injury, dental injury, chronic pain, wound complication, or infection. OMS did have a significantly higher rate of complications, due to hardware failure (9.8%), than PRS (0%) and ENT (0%) (*p* = 0.032) (Table 6).

## 4. Discussion

Mandibular fractures are one of the most common maxillofacial fractures, with epidemiological studies estimating they compose between 18.0 and 72.9% of all facial fractures [5,25,26]. The treatment of mandibular fractures is multifaceted and incorporates many specialties, including Oral and Maxillofacial Surgery (OMS), Plastic and Reconstructive Surgery (PRS), and Otolaryngology (ENT), among others [2,5]. Though common, mandibular fractures continue to have a broad range of complications and present a significant healthcare burden, with the average cost of $38,804 per patient, and complications adding a mean of $11,637 per patient [27]. The quality of evidence supporting particular treatment modalities is poor, therefore contributing to the continued variation in treatment approaches [28]. This study evaluates the management strategies of isolated mandibular fractures and assesses the effect on clinically significant outcomes among operating services at our institution. With a consistent rotating schedule, faculty roster, and access to long-term evaluation of outcomes, this study design allowed for a detailed comparison of mandibular fracture management between three operating services, which has not been feasible in previously published reports [5,13,29].

In line with the literature, our study showed that mandibular fractures occur most commonly in males (95.3%), with peak incidence in the second to third decades of life (mean age 30.6 ± 12.8) [30]. Fractures were most commonly caused by assault (79.1%), followed by falls (9.9%), in this urban location. Though consistent with previous studies [1], geographic location has also influenced the causes of mandibular fractures. Additional studies have reported traffic accidents as the most common cause of mandibular fracture in their study population [9,26], while this etiology contributed to less than 1.0% of our study population, likely due to the reduced speed limits enforced in New York City. The fractures assessed in this study were most commonly in multiple locations of the mandible (47.6%), followed by those of the angle/ramus (31.4%). This continued to be true when all fractures were evaluated individually. Combined mandibular fractures have been reported as the most common (36.7%), followed by angle of mandible fractures (24.1%) [3]. Interestingly, a study from between 1948 and 1974 at the same center as the one in this study also showed fractures of the mandibular angle as one of the most common injuries [31]. It is important to note, however, that the etiology of trauma can play a role in the fracture pattern, and the stratification of fracture location can differ among cohorts of patients [1]. Interestingly, in a study comparing battle injuries with civilian trauma, fractures of the mandibular body and angle were significantly higher in the battle-injured population than civilians, exemplifying the variety of patient groups that must be assessed individually [32].

Fracture management is subsequently affected by the diversity of fracture patterns and patient demographics [2]. Provider specialty appears to be another contributing factor in fracture management, as the observed variation suggests that clinical background may influence the decision to pursue operative versus non-operative management of fractures. This was also further influenced by location of the fracture. For example, ENT tended to opt for non-operative management more often than other specialties. Interestingly, a national surgical database found that ENT was the service providing predominantly operative treatment of mandibular fractures. However, this study did not have representation from OMS in their database, nor a relative comparison to the non-operative case load, which is central to our findings [29].

It is not surprising that decisions on mandibular fracture management differ, given that there is poor-quality evidence to support the superiority of one approach over the other [28]. There is further variability within operative management, where treatment teams can decide not only between surgical approaches, but also between ORIF alone versus ORIF with maxillomandibular fixation (MMF). When the decision was made for surgical management, all three surgical specialties in this study were more likely to access the fracture transorally (83.0%), over other surgical approaches. There was, however, a significant difference in the decision for ORIF alone versus ORIF with MMF. OMS and PRS both favored ORIF alone. This does not seem to be influenced by the number of plates used, as there was no difference for this variable between specialties (*p* = 0.230). In a questionnaire survey on postoperative intermaxillary fixation in mandibular trauma, treating surgeons identified that the most prominent reason for continuing fixation postoperatively was surrounding the concern of occlusion, with most answering that it maintained more favorable occlusion, despite the lack of supporting evidence [33].

Reducing complications is a driver of treatment strategy. Several complications have been recorded following mandibular fracture treatment. These include infection, dehiscence, malocclusion, nerve injury, malunion, non-union, hardware failure, and TMJ dysfunction, among others [6,34]. Wound complications (10.2%), including dehiscence, were the most common in this study. The infection rate was 8.8%, and comparable to previous reports [6]. Fracture characteristics [6,7,8], treatment approaches [6,9], and patient characteristics, such as substance use and chronic medical conditions, have been shown to affect complication rates to varying degrees [9,10,11,12]. This study focused on the influence of surgical specialty and found no significant difference in the overall complications, which is consistent with prior studies comparing plastic surgeons to non-plastic surgeons [5], as well as those comparing plastic surgery and otolaryngology [13]. These findings support management models that emphasize interdisciplinary collaboration and cross-specialty coverage, rather than reliance on a single specialty approach. They also emphasize the cross-disciplinary competency for management of mandibular fractures. Individually, however, this study found a significantly higher rate of hardware failure in the OMS group, without an overall statistical difference in the number of plates used per fracture. It is important to consider whether this was influenced by chart documentation, given that both PRS and ENT did not have any reports of this complication. Though these data are on the higher end of the reported overall complications (37.2%), it must not be taken lightly that this is an extensive evaluation of complications with a considerable length of follow-up for clinically significant outcomes [6].

This study is not without its limitations. As a retrospective chart review comprising over ten years, this study is subject to methodological limitations, particularly variability in documentation and the development of electronic medical records, which influenced the availability of patient information. This resulted in the exclusion of 54 patients, for whom long-term follow-up was not available due to the absence of patient charts. For those with charts available, it was assumed that absence of a documented complication equated to no complication occurring. Though retrospective studies must work under these assumptions, it is recognized that other reasons for the absence of documented complications could be due to poor documentation of events. Conversely, over-reporting may also occur due to documentation being performed by providers of multiple levels of experience. Furthermore, as another factor in retrospective studies, the authors recognize that groups are not always perfectly balanced, though the clinical services followed a consistent, evenly distributed call schedule every third day. Surgeon-specific decision-making could have also introduced unmeasured variability. When patients with multiple fractures were grouped together, the ENT group had significantly more open (*p* = 0.003) and parasymphyseal fractures (*p* = 0.025). However, when analyzing all fractures individually, the differences in fracture location were no longer significant (*p* = 0.262). There were also statistically less preoperative malocclusion documented (*p* < 0.001). This could be a limitation of documentation versus actual exam findings. The groups were not significantly different in any other baseline fracture or patient characteristic, including comorbidities and the severity of injuries (*p* > 0.05). Lastly, as with all studies, patients are often lost to follow-up, and treatment is dependent on a patient’s ability to adhere to recommendations. This may skew outcome data, which may not reflect the true complication rate. Patient compliance has been a point of discussion, particularly in patients who experience facial fractures [18]. It is therefore important that future studies address these limitations by standardizing documentation and establishing communication between treating specialties to develop a treatment protocol that can best serve our patients. Future investigation can also expand this analysis to all facial fractures, as this study is limited to isolated mandibular fractures. Additionally, multivariate models can be incorporated in larger data-sets to adjust for potential confounders, as well as to better understand the predictors of mandibular fracture management across specialties.

## 5. Conclusions

This study evaluated the overall management trends amongst three surgical services treating isolated mandibular fractures. It was found that services differ in their decision to pursue operative versus non-operative management, as well as the decision for postoperative MMF, though they agree to most commonly use the transoral approach. Overall complications were not significantly affected by this difference in decision-making. Future efforts should focus on developing a prospective, evidence-based protocol, and on expanding this analysis to all facial fractures.

## Figures and Tables

**Table 1:** Demographics and Clinical Characteristics of Patients with Mandibular Fractures.

Variable (*N* = 191)	*n* (%)
Age (mean ± SD)	30.6 ± 12.8
Male	182 (95.3)
Smoker	42 (22.0)
Major psychiatric illness	27 (14.1)
Asthma	22 (11.5)
Substance use disorder	17 (8.9)
Cardiovascular disease	10 (5.2)
Alcohol use disorder	8 (4.2)
Diabetes	5 (2.6)
**ASA Class**	
Lower than 3	182 (95.3)
3 or higher	9 (4.7)
**Etiology of Injury**	
Assault	151 (79.1)
Fall	19 (9.9)
Fight/Brawl	7 (3.7)
Sports	5 (2.6)
Pedestrian struck	3 (1.6)
Bicyclist	3 (1.6)
Aircraft or motor vehicle accident	2 (1.0)
Gunshot wound	1 (0.5)
**Surgical specialty (No. of cases)**	
OMS	86 (45.0)
PRS	54 (28.3)
ENT	51 (26.7)

OMS, Oral and Maxillofacial Surgery; PRS, Plastic and Reconstructive Surgery; ENT, Ear, Nose, and Throat—Otolaryngology; No., number

**Table 2:** Mandibular Fracture Location by Treating Specialty.

Location (*N* = 191)	*n* (%)	OMS	PRS	ENT	*p*-Value
Symphyseal	1 (0.5)	1 (1.2)	0 (0.0)	0 (0.0)	**0.025**
Parasymphyseal	10 (5.2)	1 (1.2)	2 (3.7)	7 (13.7)	
Body	14 (7.3)	7 (8.1)	3 (5.6)	4 (7.8)	
Angle/Ramus	60 (31.4)	25 (29.1)	24 (44.4)	11 (21.6)	
Condylar	3 (1.6)	2 (2.3)	1 (1.9)	0 (0.0)	
Subcondylar	12 (6.3)	3 (3.5)	5 (9.3)	4 (7.8)	
Multiple fractures	91 (47.6)	47 (54.7)	19 (35.2)	25 (49.0)	

*p*-Values calculated using Fisher’s exact test. OMS, Oral and Maxillofacial Surgery; PRS, Plastic and Reconstructive Surgery; ENT, Ear, Nose, and Throat—Otolaryngology. *p* value was bolded to indicate significance, *p* < 0.05.

**Table 3:** Mandibular Fracture Location with Multiple Fractures Individually Defined.

Location (*N* = 291)	*n* (%)	OMS	PRS	ENT	*p*-Value
Symphyseal	9 (3.1)	6 (4.3)	1 (1.3)	2 (2.6)	0.262
Parasymphyseal	50 (17.2)	20 (14.5)	10 (13.3)	20 (25.6)	
Body	50 (17.2)	27 (19.6)	10 (13.3)	13 (16.7)	
Angle/Ramus	127 (43.6)	57 (41.3)	39 (52.0)	31 (39.7)	
Coronoid	2 (0.7)	0 (0.0)	2 (2.7)	0 (0.0)	
Condylar	13 (4.5)	9 (6.5)	2 (2.7)	2 (2.6)	
Subcondylar	40 (13.7)	19 (13.8)	11 (14.7)	10 (12.8)	

*p*-Values calculated using Fisher’s exact test. OMS, Oral and Maxillofacial Surgery; PRS, Plastic and Reconstructive Surgery; ENT, Ear, Nose, and Throat—Otolaryngology.

**Table 4:** Proportion of Fractures Managed Operatively and Analysis of Fracture Location for Operative Fractures.

Variable (*N*, Total = 291; *N*, Operative = 148)	*n* (%)	OMS	PRS	ENT	*p*-Value
Total Operative (vs. Non-operative)	77.5%	88.4%	79.6%	56.9%	**<0.001**
Symphyseal	1 (0.7)	1 (1.3)	0 (0.0)	0 (0.0)	**0.002**
Parasymphyseal	8 (5.4)	1 (1.3)	1 (2.3)	6 (20.7) *	
Body	7 (4.7)	5 (6.6)	2 (4.7)	0 (0.0)	
Angle/Ramus	53 (35.8)	23 (30.3)	23 (53.5)	7 (24.1)	
Condylar	0 (0.0)	0 (0.0)	0 (0.0)	0 (0.0)	
Subcondylar	4 (2.7)	2 (2.6)	2 (4.7)	0 (0.0)	
Multiple fractures	75 (50.7)	44 (57.9)	15 (34.9)	16 (55.2)	

* Within operative fractures, ENT treated significantly more parasymphyseal fractures than OMS and PRS (*p* < 0.001). *p*-Values calculated using Chi-square test for operative management and Fisher’s exact test for fracture location. OMS, Oral and Maxillofacial Surgery; PRS, Plastic and Reconstructive Surgery; ENT, Ear, Nose, and Throat—Otolaryngology. *p* value was bolded to indicate significance, *p* < 0.05.

**Table 5:** Mandibular Fracture Surgical Approach by Specialty.

	*n* (%)	OMS	PRS	ENT	*p*-Value
ORIF Alone	93 (62.8)	53 (69.7)	30 (69.8)	10 (34.5)	**0.002**
ORIF + MMF	55 (37.2)	23 (30.3)	13 (30.2)	19 (65.5)	
Transoral only	122 (83.0)	59 (78.7)	35 (81.4)	28 (96.6)	0.211
Transfacial only	14 (9.5)	10 (13.3)	4 (9.3)	0 (0.0)	
Combination	11 (7.5)	6 (8.0)	4 (9.3)	1 (3.4)	
Mean number of plates per fracture	1.20 ± 0.52	1.15 ± 0.51	1.29 ± 0.50	1.24 ± 0.58	0.230

Mean number of plates per fracture is documented as Mean ± Standard Deviation. *p*-Values calculated using Chi-square test for ORIF or ORIF + MMF, Fisher’s exact test for operative approach, and one-way ANOVA for plates per fracture. OMS, Oral and Maxillofacial Surgery; PRS, Plastic and Reconstructive Surgery; ENT, Ear, Nose, and Throat—Otolaryngology; ORIF, open reduction-internal fixation; MMF, maxillomandibular fixation. *p* value was bolded to indicate significance, *p* < 0.05.

**Table 6:** Clinically Significant Complications and Follow-Up in Clinic.

Variable (*N* = 137)	*n* (%)	OMS (*n* = 61)	PRS (*n* = 37)	ENT (*n* = 39)	*p*-Value
**Complication**					
New malocclusion	13 (9.5)	9 (14.8)	2 (5.4)	2 (5.1)	0.223
Aesthetic deformity	4 (2.9)	3 (4.9)	1 (2.7)	0 (0.0)	0.384
TMJ Dysfunction	1 (0.7)	0 (0.0)	1 (2.7)	0 (0.0)	0.270
New nerve injury	13 (9.5)	8 (13.1)	4 (10.8)	1 (2.6)	0.193
Hardware failure	6 (4.4)	6 (9.8)	0 (0.0)	0 (0.0)	**0.032**
Dental injury	2 (1.5)	1 (1.6)	1 (2.7)	0 (0.0)	0.745
Chronic pain	10 (7.3)	6 (9.8)	1 (2.7)	3 (7.7)	0.490
Wound complication	14 (10.2)	3 (4.9)	3 (8.1)	8 (20.5)	0.053
Infection	12 (8.8)	5 (8.2)	2 (5.4)	5 (12.8)	0.507
**Overall Complication Rate**	51 (37.2)	27 (44.3)	10 (27.0)	14 (35.9)	0.227
**Length of follow-up (days)**	49.0 (43.0–58.0)	63.0 (47.0–75.0)	30.0 (13.0–46.0)	48.0 (34.0–85.0)	0.899

Length of follow-up is documented as Median (95% Confidence Interval). *p*-Values calculated using Fisher’s exact test for individual complications, Chi-square test for overall complication rate, and Kruskal–Wallis test used for length of follow-up. OMS, Oral and Maxillofacial Surgery; PRS, Plastic and Reconstructive Surgery; ENT, Ear, Nose, and Throat—Otolaryngology; TMJ, temporomandibular joint. *p* value was bolded to indicate significance, *p* < 0.05.

## Data Availability

Data are contained within the article. The original contributions presented in this study are included in the article. Further inquiries can be directed to the corresponding author.
